# *Ganoderma lucidum* polysaccharides counteract inhibition on CD71 and FasL expression by culture supernatant of B16F10 cells upon lymphocyte activation

**DOI:** 10.3892/etm.2013.931

**Published:** 2013-01-29

**Authors:** LI-XIN SUN, ZHI-BIN LIN, XIN-SUO DUAN, JIE LU, ZHI-HUA GE, MIN LI, EN-HONG XING, TIAN-FEI LAN, MIAO-MIAO JIANG, NING YANG, WEI-DONG LI

**Affiliations:** 1 The Affiliated Hospital of Chengde Medical College, Chengde, Hebei 067000;; 2 Department of Pharmacology, Peking University Health Science Center,School of Basic Medical Sciences, Beijing 100191, P.R. China

**Keywords:** tumor, lymphocyte activation, *Ganoderma lucidum*, polysaccharides, FasL, CD71

## Abstract

Immune responses to tumor-associated antigens are often detectable in tumor-bearing hosts, but they fail to eliminate malignant cells or prevent development of metastases. Tumor cells produce factors such as interleukin-10, transforming growth factor-β1 and vascular endothelial growth factor (VEGF) that suppress the function of immune cells or induce apoptosis of immune cells. Culture supernatant of tumor cells may contain these immunosuppressive factors which suppress lymphocyte activation. CD71 and FasL are two important molecules that are expressed upon lymphocyte activation. Counteraction against suppression CD71 and FasL expression upon lymphocyte activation may benefit tumor control. A potential component with this effect is *Ganoderma lucidum* polysaccharides (*Gl*-PS). In this study, *Gl*-PS was used on lymphocytes incubating with culture supernatant of B16F10 melanoma cells (B16F10-CS) in the presence of phytohemagglutinin. Following induction with phytohemagglutinin, B16F10-CS suppressed CD71 expression in lymphocytes (as detected by immunofluorescence and flow cytometry), proliferation in lymphocytes (as detected by MTT assay), and FasL expression in lymphocytes (as detected by immunocytochemistry and western blot analysis), while *Gl*-PS fully or partially counteracted these suppressions. *Gl*-PS showed counteractive effects against suppression induced by B16F10-CS on CD71 and FasL expression upon lymphocyte activation, suggesting the potential of *Gl*-PS to facilitate cancer immunotherapy.

## Introduction

A number of findings in both mouse models of cancer and humans with cancer provide compelling evidence that, as a primary defense against cancer, the immune system specifically identifies and eliminates tumor cells on the basis of their expression of tumor-specific antigens or molecules induced by cellular stress (1). Nevertheless, various attempts to perform specific and nonspecific immunotherapy for human cancer in clinical trials have shown limited or no success (2). The immune suppression directly or indirectly induced by tumor cells may account in part for the reasons. Cancer cells display multiple immunosuppressive mechanisms to evade T-cell responses, either to avoid immune recognition or to disable effector T-cells (3). They produce and release factors to suppress the function or induce the apoptosis of immune cells (4). B16F10 cells as a melanoma cell line derived from C57BL mice may also produce and release the immune suppressing factors. The culture supernatant of B16F10 cells (B16F10-CS) may, therefore, contain the immune suppressing factors and thereby have the effects of suppression on lymphocyte activation. Antagonism of the suppression of lymphocyte activation may benefit tumor control, and *Ganoderma lucidum* polysaccharides (*Gl*-PS), with its multiple bioactivities may have this potential.

*Gl*-PS is the critical bioactive component of *Ganoderma lucidum* (*Gl*), a type of fungus commonly known as Lingzhi or Reishi mushroom which has been widely used as medicine to promote health and longevity in China for thousands of years (5,6). It has been shown that many natural products have immunomodulatory and antitumor effects (7–15), as well as *Gl*-PS. The multiple biological activities of *Gl*-PS include improvement of host immune function (16), prevention of oxidative damage (17), protection of liver and reduction of serum glucose levels, along with a lack of toxicity (18,19), modification of biological response and potentiation of immune effectiveness (20). Previous studies demonstrated the effects of *Gl*-PS on acceleration of wound repair in intestinal epithelial cells (21), antagonism against the tumor-induced immunosuppression (22), promotion of B16F10 cells to activate lymphocytes (23), induction of stronger cytotoxicity in cytotoxic lymphocytes (CTLs) with granzyme B and porforin by action on B16F10 cells (24), and enhancement of major histocompatability complex (MHC) class I and costimulatory molecules on B16F10 cells to induce stronger anti-B16F10 cytotoxicity in lymphocytes (25). Nevertheless, the effects of *Gl*-PS on the counteraction against the suppression of CD71 and FasL expression upon lymphocyte activation induced by B16F10-CS remain to be confirmed. In the present study, a mouse melanoma cell line, the B16F10 cell, was used to evaluate the role of *Gl*-PS on the counteraction against the suppression induced by B16F10-CS of CD71 and FasL expression upon lymphocyte activation.

## Materials and methods

### Animals and drugs

Inbred strain C57BL/6 (H-2^b^) mice were purchased from the Department of Experimental Animals, Health Science Centre, Peking University, Beijing, China. The use of mice was approved by the ethics committee of The Affiliated Hospital of Chengde Medical College (Chengde, China). As previously described (26), *Gl*-PS was isolated from the boiling water extract of the fruit bodies of *Gl* by ethanol precipitation and dialysis, followed by deproteination with Sevag. The molecular weight of the *Gl*-PS was 584,900 Da, with a ratio of polysaccharides to peptides of 93.61:6.49. The polysaccharides were composed of D-rhamnose, D-xylose, D-fructose, D-galactose, D-mannose, D-glucose and uronic acid. The *Gl*-PS as water-soluble powder was dissolved in the B16F10-CS, filtered through a 0.22 *μ*m filter and stored at 4°C before use.

### Preparation of the B16F10-CS

As described previously, the B16F10-CS was prepared with B16F10 melanoma cells. Mouse B16F10 cells were grown at 37°C in a humidified atmosphere containing 5% CO*_2_* in RPMI-1640 medium supplemented with 10% fetal bovine serum (FBS), penicillin (100 IU/ml) and streptomycin (100 *μ*g/ml). The B16F10 cells were cultured in 6-well culture plates (1×10^5^ cells/well at the start). The RPMI-1640 medium was replaced with a fresh solution when 80% confluency was reached, and incubated for a further 8 h. The supernatants of the cultures (B16F10-CS) were harvested, filtered through a 0.22-*μ*m filter and stored at 4°C.

### Preparation of splenic mononuclear lymphocytes

As described previously (22), mouse mononuclear lymphocytes were isolated from splenocytes of C57BL/6 mice in a Ficoll-Urografin density gradient and counted by light microscopy with few cells nonviable. Cells were placed into the wells of 96-well flat-bottomed microplates at 1×10^6^ cells/well, with the B16F10-CS containing different concentrations of *Gl*-PS (0.2, 0.8, 3.2 and 12.8 *μ*g/ml) and 20 *μ*g/well of phytohemagglutinin (PHA). The total volume of each well was 200 *μ*l. Two controls were RPMI-1640 medium alone (containing neither B16F10-CS nor *Gl*-PS) and B16F10-CS alone (without *Gl*-PS). The cells were cultured for 48 h for detection of CD71 (transferrin receptor) expression and 72 h for detection of cell proliferation as well as FasL expression.

### Assay of splenic mononuclear lymphocyte proliferation

Cell proliferation was measured by 3-[4,5-dimethylthiazol-2-yl]-2,5-diphenyltetrazolium bromide (MTT) assay after 72 h incubation (27). A total of 20 *μ*l (5 mg/ml) MTT (Sigma, St. Louis, MO, USA) solution was added to each well 4 h before termination. After 4 h incubation, the cells were lysed and the purple formazan crystals were solubilized for detection of the optical density (OD) at 490 nm.

### Determination of CD71 expression by immunofluorescence staining and flow cytometry

The immunofluorescence staining was performed as described previously (23). After 48 h incubation, splenic mononuclear lymphocytes were washed twice with washing buffer [2% FBS in phosphate-buffered saline, (PBS)]. Serum of mice and purified rat anti-mouse CD16/CD32 antibody (BD Pharmingen, San Jose, CA, USA) were used to block non-specific antibody binding. PE-conjugated anti-mouse CD71 (Beckman Coulter, Inc., Fullerton, CA, USA) antibody was added to the cells and left at 4°C for 45 min in the dark. The cells were then mounted on slides, and examined and photographed under a fluorescence microscope. Replacement of primary antibody with PBS was used as a negative control. Cells were also determined by flow cytometry analysis. Fluorescence profiles were generated on a FACSCalibur flow cytometer (Becton Dickinson, San Jose, CA, USA). Histogram and density plots were produced by the CellQuest software package (Becton Dickinson). Dead cells and debris were gated out.

### Determination of FasL production by immunocytochemistry assay

The immunocytochemistry assay was performed as described previously (22). After 24 h incubation, splenic mono-nuclear lymphocytes were smeared on slides and fixed with cold acetone for 5 min. The endogenous peroxidase activity was quenched with 3% hydrogen peroxide. After blocking with 10% normal serum, goat primary antibody against FasL (Santa Cruz Biotechnology, Inc., Santa Cruz, CA, USA) was used at a 1:50 dilution and incubated overnight at 4°C. The next day, the horseradish peroxidase (HRP)-labeled secondary antibody was applied for 1 h and staining was finalized with a diaminobenzidine (DAB) solution to detect the target antigen. Slides were extensively washed with PBS between the different stages and counterstained with hematoxylin before mounting. Slides were examined and photographed under a light microscope. Replacement of primary antibody with PBS was used as a negative control.

### SDS-polyacrylamide electrophoresis and western blot analysis

The protein levels of FasL in splenic mononuclear lymphocytes were determined by western blot analysis. The levels of total protein extracted from the splenic mononuclear lymphocytes were determined with the Bradford assay. Equal amounts of protein (50 *μ*g) were subjected to SDS-PAGE and transferred to PVDF membranes. The membranes were subsequently pre-blocked in TBS containing 5% non-fat milk powder and then incubated with mouse polyclonal anti-FasL antibody (Santa Cruz Biotechnology, Inc.) at a dilution of 1:100 followed by peroxidase-conjugated secondary antibody for detection. The antigen-antibody complex was visualized with western blot analysis luminol reagent (Santa Cruz Biotechnology, Inc.). The bands were quantified with a Gel Doc 2000 system and Quantity One software (Bio-Rad, Hercules, CA, USA) and expressed as a ratio (FasL vs. β-actin) followed by standardization.

### Statistical analysis

The results, except immunofluorescence and immunocytochemistry, are expressed as the mean (± SD) of triplicate experiments in western blot analysis, or octuplus experiments in cell proliferation assay with MTT, and statistical comparison between the experimental groups versus the control was performed using one-way ANOVA, followed by the Dunnett’s t-test. P<0.05 was considered to indicate a statistically significant result.

## Results

### Suppression by B16F10-CS and antagonism by Gl-PS on CD71 expression in splenic mononuclear lymphocytes induced by PHA

**Figure 1:**
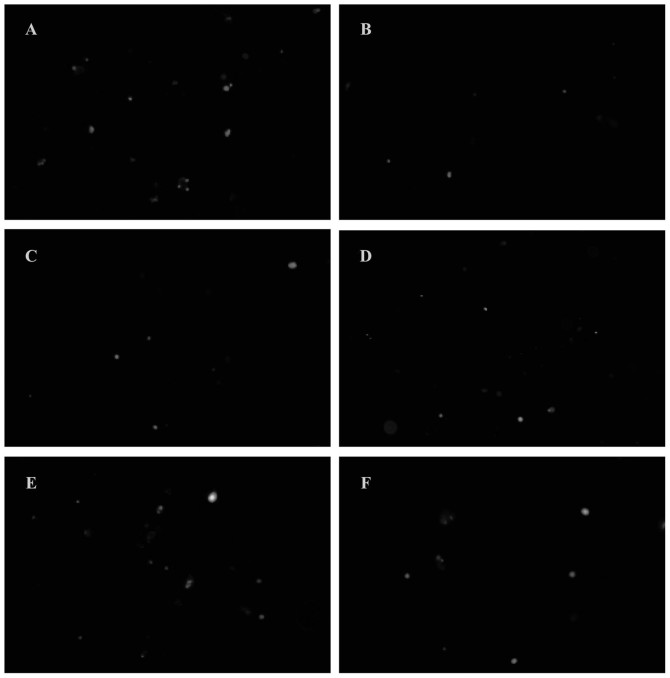
Suppression by B16F10-CS and counteraction by *Ganoderma lucidum* polysaccharides (*Gl*-PS) on CD71 expression in splenic mono-nuclear lymphocytes induced by phytohemagglutinin (PHA) measured by immunofluorescence. After 48 h incubation, the responses of CD71 expression in splenic mononuclear lymphocytes to PHA were shown by immunofluorescence. (A) Control wells containing neither B16F10-CS nor *Gl*-PS. (B) Wells containing B16F10-CS. (C) Wells containing B16F10-CS and 0.2 *μ*g/ml *Gl*-PS. (D) Wells containing B16F10-CS and 0.8 *μ*g/ml *Gl*-PS. (E) Wells containing B16F10-CS and 3.2 *μ*g/ml *Gl*-PS. (F) Wells containing B16F10-CS and 12.8 *μ*g/ml Gl-PS.

**Figure 2:**
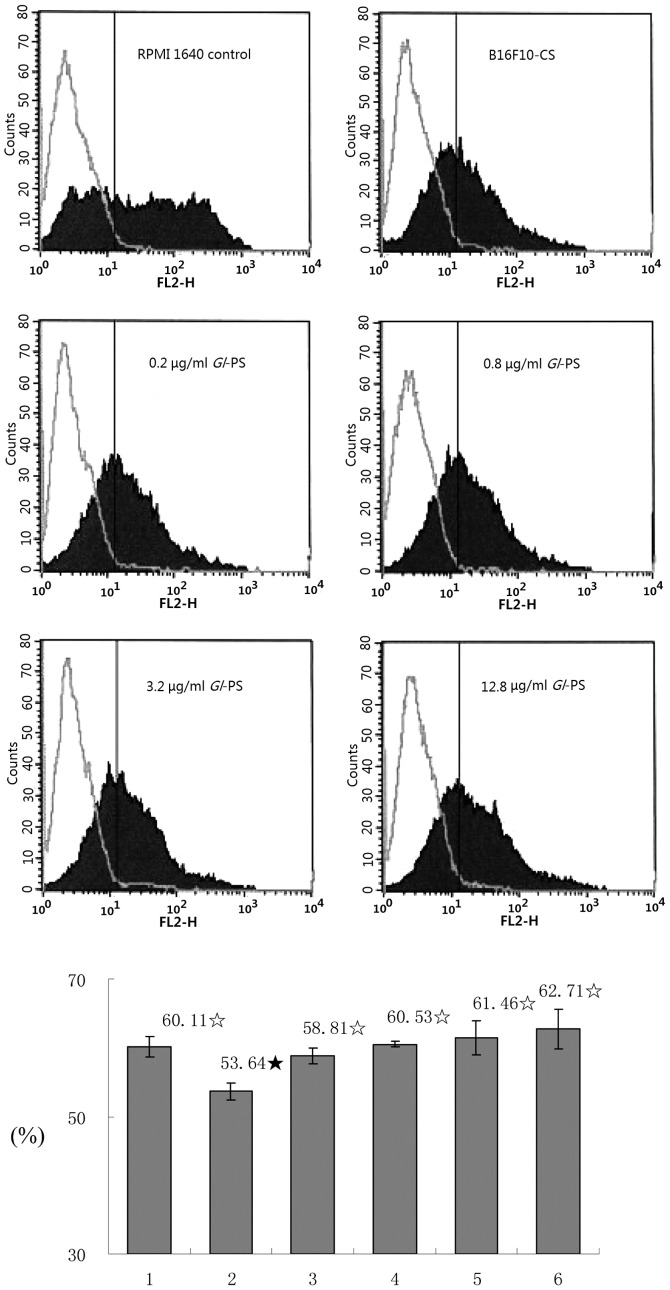
Suppression by B16F10-CS and counteraction by *Ganoderma lucidum* polysaccharides (*Gl*-PS) on CD71 expression in splenic mono-nuclear lymphocyte induced by phytohemagglutinin (PHA) measured by flowcytometry. After 48 h incubation, the rate of CD71 expressing cells in splenic mononuclear lymphocytes to PHA was shown by flowcytometry. (1) Control wells containing neither B16F10-CS nor Gl-PS. (2) Wells containing B16F10-CS. (3) Wells containing B16F10-CS and 0.2 *μ*g/ml *Gl*-PS. (4) Wells containing B16F10-CS and 0.8 *μ*g/ml Gl-PS. (5) Wells containing B16F10-CS and 3.2 *μ*g/ml *Gl*-PS. (6) Wells containing B16F10-CS and 12.8 *μ*g/ml Gl-PS. Open histograms represent the isotype control. Error bars indicate the SD. ^★^P<0.05, significantly different compared with the RPMI 1640 medium control (with neither B16F10-CS nor Gl-PS); ^⋆^P<0.05, significantly different compared with the B16F10-CS control (without *Gl*-PS); one-way analysis of variance followed by Dunnett’s t-test.

During the activation of lymphocytes, some cluster of differentiation (CD) appears which may serve as activation markers, including early (CD69 and CD71) and late (CD25, CD26, HLA//DR) activation markers (28). Therefore, in addition to CD69, CD71 is an early activation marker which is useful in evaluating lymphocyte activation responses to antigens or mitogens. The suppression of lymphocyte activation may result in the suppression of CD71 expression. CD71 expression was detected on lymphocytes. The lymphocytes were stimulated with PHA and cultured with B16F10-CS and *Gl*-PS for 48 h. It was shown by immunofluorescence that the CD71 expression in mononuclear lymphocytes was reduced after 48 h induction by PHA in B16F10-CS wells (without *Gl*-PS), when compared with the RPMI-1640 medium control, while *Gl*-PS in the wells antagonized the reduction of the CD71 expression (Fig. 1). It was also shown by flow cytometry that the expression rate of CD71 in the lymphocytes was much lower in B16F10-CS wells (without *Gl*-PS) than that in RPMI-1640 medium control wells (P<0.05). The expression rate was higher in the wells with B16F10-CS and any amount of *Gl*-PS used in this study than that in wells with B16F10-CS without *Gl*-PS (all P<0.05, Fig. 2), showing the antagonism of *Gl*-PS on the reduction by B16F10-CS on the CD71 expression in lymphocytes after induction by PHA.

### Suppression by B16F10-CS and antagonism by Gl-PS of splenic mononuclear lymphocyte proliferation induced by PHA

**Figure 3:**
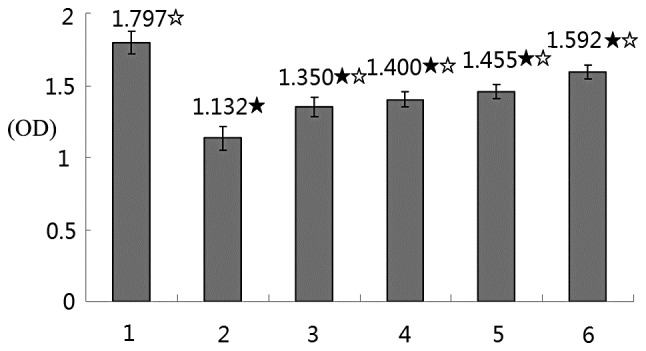
Suppression by B16F10-CS and counteraction by *Ganoderma lucidum* polysaccharides (*Gl*-PS) on splenic mononuclear lymphocyte proliferation induced by phytohemagglutinin (PHA). After 72 h incubation, the proliferative responses of splenic mononuclear lymphocytes to PHA were measured by MTT assay. (1) Control wells containing neither B16F10-CS nor *Gl*-PS. (2) Control wells containing B16F10-CS. (3) Wells containing B16F10-CS and 0.2 *μ*g/ml *Gl*-PS. (4) Wells containing B16F10-CS and 0.8 *μ*g/ml *Gl*-PS. (5) Wells containing B16F10-CS and 3.2 *μ*g/ml *Gl*-PS. (6) Wells containing B16F10-CS and 12.8 *μ*g/ml *Gl*-PS. Error bars indicate the SD. ^★^P<0.05, significantly different compared with the RPMI-1640 medium control (with neither B16F10-CS nor *Gl*-PS); ^⋆^P<0.05, significantly different compared with the B16F10-CS control (without *Gl*-PS); one-way analysis of variance followed by Dunnett’s t-test.

The activation of the lymphocytes can be disclosed by proliferation induced by mitogens such as PHA. Therefore, suppression of cell proliferation may reflect the suppression of lymphocyte activation. The lymphocyte proliferation by MTT assay after PHA stimulation was also detected. Compared with the RPMI-1640 medium control, 72 h after induction with PHA, the OD in B16F10-CS wells (without *Gl*-PS) was markedly reduced (P<0.05), while in the wells containing different concentrations of *Gl*-PS, the reduction of the OD was antagonized significantly (P<0.05, Fig. 3).

### Suppression by B16F10-CS and antagonism by Gl-PS of FasL expression in splenic mononuclear lymphocytes induced by PHA

**Figure 4:**
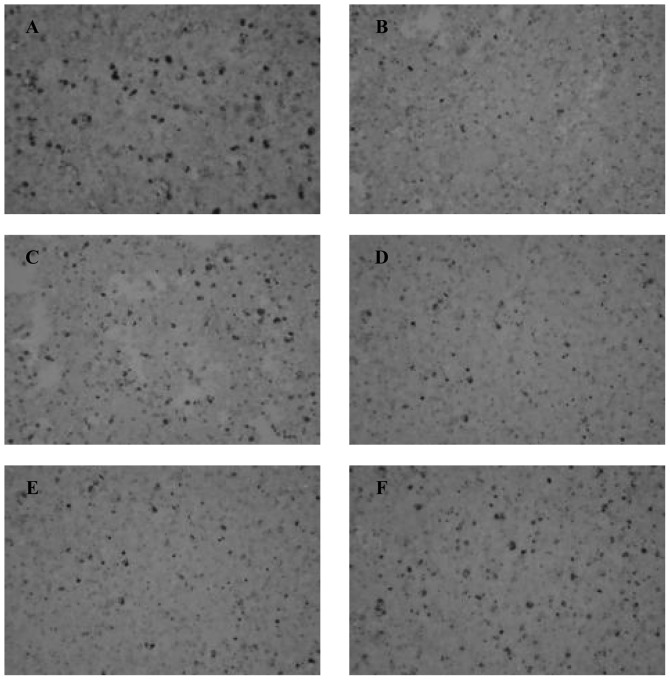
Suppression by B16F10-CS and counteraction by *Ganoderma lucidum* polysaccharides (*Gl*-PS) on FasL expression in splenic mononuclear lymphocytes induced by phytohemagglutinin (PHA) for 72 h measured by immunocytochemistry. (A) Control wells containing neither B16F10-CS nor *Gl*-PS. (B) Control wells containing B16F10-CS. (C) Wells containing B16F10-CS and 0.2 *μ*g/ml *Gl*-PS. (D) Wells containing B16F10-CS and 0.8 *μ*g/ml *Gl*-PS. (E) Wells containing B16F10-CS and 3.2 *μ*g/ml *Gl*-PS. (F) Wells containing B16F10-CS and 12.8 *μ*g/ml *Gl*-PS.

**Figure 5:**
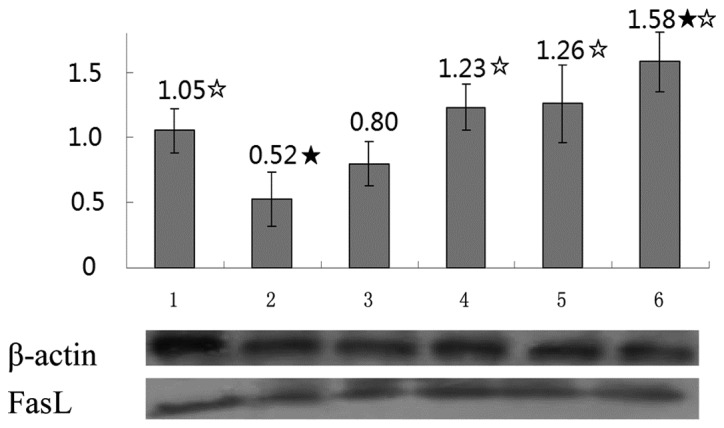
Suppression by B16F10-CS and counteraction by *Ganoderma lucidum* polysaccharides (*Gl*-PS) on FasL expression in splenic mononuclear lymphocyte induced by phytohemagglutinin (PHA). After 72 h incubation, the FasL expression in splenic mononuclear lymphocytes responses to PHA was measured by western blot analysis. (1) Control wells containing neither B16F10-CS nor *Gl*-PS. (2) Control wells containing B16F10-CS. (3) Wells containing B16F10-CS and 0.2 *μ*g/ml *Gl*-PS. (4) Wells containing B16F10-CS and 0.8 *μ*g/ml *Gl*-PS. (5) Wells containing B16F10-CS and 3.2 *μ*g/ml *Gl*-PS. (6) Wells containing B16F10-CS and 12.8 *μ*g/ml *Gl*-PS. The experiment was performed in triplicate and the means and SD were calculated and showed in the histogram. Error bars indicate the SD. ^★^P<0.05, significantly different compared with the RPMI-1640 medium control (with neither B16F10-CS nor *Gl*-PS); ^⋆^P<0.05, significantly different compared with the B16F10-CS control (without *Gl*-PS); one-way analysis of variance followed by Dunnett’s t-test.

FasL expression appears upon lymphocyte activation for the maintenance of immune homeostasis as well as a pathway of killing target cells. The FasL expression in splenic mononuclear lymphocytes induced by mitogen such as PHA is also closely associated with the activation of the lymphocytes to kill target cells. The suppression of lymphocyte activation may, therefore, lead to the suppression of FasL expression. The FasL expression in the lymphocytes was detected. Immunocytochemistry showed that FasL expression in mononuclear lymphocytes was markedly reduced after 24 h incubation with PHA in B16F10-CS wells (without *Gl*-PS), when compared with the RPMI-1640 medium control. *Gl*-PS in the wells antagonized the reduction of the FasL expression (Fig. 4). Western blot analysis showed that FasL expression in mononuclear lymphocytes induced by PHA was markedly reduced after 72 h in B16F10-CS wells (without *Gl*-PS), when compared with the RPMI-1640 medium control (P<0.05), while 0.8, 3.2 and 12.8 *μ*g/ml *Gl*-PS statistically antagonized the reduction of FasL expression (P<0.05, Fig. 5).

## Discussion

Immune responses to tumor-associated antigens (TAs) are often detectable in tumor-bearing hosts, but they fail to eliminate malignant cells or prevent the development of metastases (29). Human tumors use many different mechanisms of immuno-suppression to prevent immune cells from exercising their antitumor activity, which enables the tumor to escape from the host’s immune system and progress (30). The production and secretion of soluble factors suppressing the functions of immune cells or inducing the apoptosis of immune cells comprise part of the immunosuppressive mechanisms (31). The IL-10, TGF-β, VEGF and prostaglandins (PGs) are common immunosuppressive factors produced by tumors which may directly or indirectly suppress the immune response and obstruct immunotherapy (32). One of the most important aims of tumor immunotherapy is, therefore, to counteract tumor-induced immunosuppression. B16F10 cells are melanoma cells derived from C57BL mice, which may produce immunosuppressive factors as well so that the B16F10-CS may have the immunosuppressive effects (22).

*Gl*-PS, the critical component of *Gl*, has multiple bio activities. In this study, the effects of *Gl*-PS on the counteraction against the suppression induced by B16F10-CS of CD71 and FasL expression upon lymphocyte activation was examined. The proliferation, CD71 expression and FasL expression were detected in the lymphocytes which were activated by PHA to determine the suppression induced by B16F10-CS and the counteraction by *Gl*-PS against the suppression.

The expression of activation markers on the cell surface is useful in evaluating the lymphocyte response to antigens or mitogens. CD71 is a lymphocyte activation marker, as are CD69 and CD25. CD71 expression on the cell surface represents the state of metabolic activity in the cell and must be increased in order to internalize the iron required for T-cell DNA synthesis and proliferation (33). It was shown in this study that CD71 expression in lymphocytes after activation by PHA was suppressed by B16F10-CS, while the suppression was antagonized by *Gl*-PS. CD71 is a transferrin receptor, a membrane receptor involved in the control of iron supply to the cell through the binding of transferrin, the major iron-carrier protein. This receptor plays a key role in the control of cell proliferation since iron is essential for sustaining ribonucleo-tide reductase activity, and is the only enzyme that catalyzes the conversion of ribonucleotides to deoxyribonucleotides (34). Suppression of the CD71 expression in lymphocytes may, therefore, lead to suppression of the proliferation and function of lymphocytes, while counteraction of this suppression by *Gl*-PS may benefit the lymphocyte activation and function.

T-cell activation is a very tightly regulated process resulting in the production of cytokines as well as clonal expansion and differentiation of effector T lymphocytes (35). Upon activation, T-cells undergo complex structural and cytoskeletal changes leading to cell cycle progression (36). The end result is the activation and expansion of a pool of effector cells that participate in cancer destruction. Proliferation is, therefore, a common feature of lymphocyte activation. It was shown in this study that after activation by PHA the proliferation of lymphocytes was suppressed by the B16F10-CS, while the suppression was counteracted by *Gl*-PS.

FasL is an inductive molecule expressed in T-cells, and weighs 40 kDa (37). It is homologous to the cytokine tumor necrosis factor (TNF), and is a member of the TNFR-II type family (38). FasL expression depends on the transcription factor levels. The positive regulators are NFAT, Egr2/Egr3, NFκB, AP-1, c-myc SP1 and B1/Cdk1, while the negative regulators are c-Fos and CIITA (39–41). Binding of Fas with FasL causes trimerization and recruitment of Fas-associated death domain (FADD) proteins through homotypic death domain interactions. In turn, trimerized FADD recruits either procaspase 8 or 10, which then becomes an activated caspase through a process of autoproteolysis (42). Assembly of these components results in the formation of a death-inducing signaling complex (DISC), which is pivotal in the receptor-dependent apoptosis (42). Caspase 8 interacts with procaspases 3, 6, or 7 and, after a process of transproteolysis, they become activated caspases. Finally, these effector caspases cleave DNA. Caspase 8 can also hydrolyze Bid, which causes damage to the mitochondrial outer membrane and triggers cytochrome-C release (43,44). FasL expression is, therefore, also an event in lymphocyte activation. In addition to maintaining immune homeostasis, FasL expressed on activated lymphocytes contributes to killing target cells by providing death signals. FasL crosslinks Fas, a death receptor expressed on the target cells, which causes the apoptosis of the target cells by triggering the apoptosis pathway (45). It was shown in this study that the expression of FasL in the lymphocytes after activation by PHA was suppressed by the B16F10-CS, while the suppression was antagonized by *Gl*-PS, especially at higher concentrations, indicating the suppression of lymphocyte activation by the B16F10-CS as well as the counteraction by *Gl*-PS.

In conclusion, counteraction against the suppression by tumor cells on CD71 expression, cell proliferation, FasL expression in lymphocytes after induction with PHA denotes a counteraction against the suppression by tumor cells on the function of lymphocytes, which is important to facilitate cancer immunotherapy, therefore, *Gl*-PS showing the effects of counteraction against the suppression induced by B16F10-CS on CD71 expression, cell proliferation, as well as FasL expression after induction with PHA, fully or partially, indicated the potential of *Gl*-PS to facilitate cancer immunotherapy.

## References

[1] Swann JB, Smyth MJ (2007). Immune surveillance of tumors. J Clin Invest.

[2] Gross S, Walden P (2008). Immunosuppressive mechanisms in human tumors: why we still cannot cure cancer. Immunol Lett.

[3] Rabinovich GA, Gabrilovich D, Sotomayor EM (2007). Immunosuppressive strategies that are mediated by tumor cells. Annu Rev Immunol.

[4] Whiteside TL (2006). Immune suppression in cancer: effects on immune cells, mechanisms and future therapeutic intervention. Semin Cancer Biol.

[5] Shao BM, Dai H, Xu W, Lin ZB, Gao XM (2004). Immune receptors for polysaccharides from *Ganoderma lucidum*. Biochem Biophys Res Commun.

[6] Chan WK, Cheung CC, Law HK, Lau YL, Chan GC (2008). *Ganoderma lucidum* polysaccharides can induce human monocytic leukemia cells into dendritic cells with immunostimulatory function. J Hematol Oncol.

[7] Amarante MK, Watanabe MA, Conchon-Costa I, Fiori LL, Oda JM, Búfalo MC, Sforcin JM (2012). The effects of propolis on CCL5 and IFN-γ expression by peripheral blood mononuclear cells from leishmaniasis patients. J Pharm Pharmacol.

[8] Kour K, Sangwan PL, Khan I, Koul S, Sharma SN, Kitchlu S, Bani S (2011). Alcoholic extract of Cicer microphyllum augments Th1 immune response in normal and chronically stressed Swiss albino mice. J Pharm Pharmacol.

[9] Sliva D (2010). Medicinal mushroom *Phellinus linteus* as an alternative cancer therapy. Exp Ther Med.

[10] Vermorken AJ, Zhu J, Van de Ven WJ, Cui Y, Fryns JP (2010). Curcumin for the prevention of progression in monoclonal gammopathy of undetermined significance: A word of caution. Exp Ther Med.

[11] Li QQ, Wang G, Reed E, Huang L, Cuff CF (2010). Evaluation of cisplatin in combination with β-elemene as a regimen for prostate cancer chemotherapy. Basic Clin Pharmacol Toxicol.

[12] Mishra N, Tiwari S, Vaidya B, Agrawal GP, Vyas SP (2011). Lectin anchored PLGA nanoparticles for oral mucosal immunization against hepatitis B. J Drug Target.

[13] Zhong Z, Dong Z, Yang L, Chen X, Gong Z (2012). Inhibition of proliferation of human lung cancer cells by green tea catechins is mediated by upregulation of let-7. Exp Ther Med.

[14] Yang Y, Jin C, Li H, He Y, Liu Z, Bai L, Dou K (2012). Improved radiosensitizing effect of the combination of etanidazole and paclitaxel for hepatocellular carcinoma in vivo. Exp Ther Med.

[15] Yang X, Hu W, Zhang Q, Wang Y, Sun L (2010). Puerarin inhibits C-reactive protein expression via suppression of nuclear factor kappaB activation in lipopolysaccharide-induced peripheral blood mononuclear cells of patients with stable angina pectoris. Basic Clin Pharmacol Toxicol.

[16] Lin ZB, Zhang HN (2004). Anti-tumor and immunoregulatory activities of *Ganoderma lucidum* and its possible mechanisms. Acta Pharmacol Sin.

[17] You YH, Lin ZB (2002). Protective effects of *Ganoderma lucidum* polysaccharides peptide on injury of macrophages induced by reactive oxygen species. Acta Pharmacol Sin.

[18] Zhang GL, Wang YH, Ni W, Teng HL, Lin ZB (2002). Hepatoprotective role of *Ganoderma lucidum* polysaccharide against BCG-induced immune liver injury in mice. World J Gastroenterol.

[19] Zhang HN, He JH, Yuan L, Lin ZB (2003). In vitro and in vivo protective effect of *Ganoderma lucidum* polysaccharides on alloxan-induced pancreatic islets damage. Life Sci.

[20] Zhu XL, Lin ZB (2006). Modulation of cytokines production, granzyme B and perforin in murine CIK cells by *Ganoderma lucidum* polysaccharides. Carbohydrate Polymers.

[21] Sun LX, Chen LH, Lin ZB, Qin Y, Zhang JQ, Yang J, Ma J, Ye T, Li WD (2011). Effects of *Ganoderma lucidum* polysaccharides on IEC-6 cell proliferation, migration and morphology of differentiation benefiting intestinal epithelium healing in vitro. J Pharm Pharmacol.

[22] Sun LX, Lin ZB, Duan XS, Lu J, Ge ZH, Li XJ, Li M, Xing EH, Jia J, Lan TF, Li WD (2011). *Ganoderma lucidum* polysaccharides antagonize the suppression on lymphocytes induced by culture supernatants of B16F10 melanoma cells. J Pharm Pharmacol.

[23] Sun LX, Lin ZB, Li XJ, Li M, Lu J, Duan XS, Ge ZH, Song YX, Xing EH, Li WD (2011). Promoting effects of *Ganoderma lucidum* polysaccharides on B16F10 cells to activate lymphocytes. Basic Clin Pharmacol Toxicol.

[24] Sun LX, Lin ZB, Duan XS, Lu J, Ge ZH, Song YX, Li XJ, Li M, Xing EH, Yang N, Li WD (2012). Stronger cytotoxicity in CTLs with granzyme B and porforin was induced by *Ganoderma lucidum* polysaccharides acting on B16F10 cells. Biomed Prev Nutr.

[25] Sun LX, Lin ZB, Duan XS, Lu J, Ge ZH, Li XF, Li XJ, Li M, Xing EH, Song YX, Jia J, Li WD (2012). Enhanced MHC class I and costimulatory molecules on B16F10 cells by *Ganoderma lucidum* polysaccharides. J Drug Target.

[26] Cao LZ, Lin ZB (2002). Regulation on maturation and function of dendritic cells by *Ganoderma lucidum* polysaccharides. Immunol Lett.

[27] Wang YY, Khoo KH, Chen ST, Lin CC, Wong CH, Lin CH (2002). Studies on the immuno-modulating and antitumor activities of *Ganoderma lucidum* (Reishi) polysaccharides: functional and proteomic analyses of a fucose-containing glycoprotein fraction responsible for the activities. Bioorg Med Chem.

[28] Reddy M, Eirikis E, Davis C, Davis HM, Prabhakar U (2004). Comparative analysis of lymphocyte activation marker expression and cytokine secretion profile in stimulated human peripheral blood mononuclear cell cultures: an in vitro model to monitor cellular immune function. J Immunol Methods.

[29] Whiteside TL (2010). Immune responses to malignancies. J Allergy Clin Immunol.

[30] Whiteside TL, Mandapathil M, Szczepanski M, Szajnik M (2011). Mechanisms of tumor escape from the immune system: adenosine-producing Treg, exosomes and tumor-associated TLRs. Bull Cancer.

[31] Wongkajornsilp A, Luankosolchai RA, Huabprasert S, Chanyavanich V, Tisavipat N, Watanapa P (2001). The observation of immunosuppressor(s) derived from cultured tumor cells and its partial neutralization with OK-432. J Med Assoc Thai.

[32] Frumento G, Piazza T, Di Carlo E, Ferrini S (2006). Targeting tumor-related immunosuppression for cancer immunotherapy. Endocr Metab Immune Disord Drug Targets.

[33] Cortés-Barberena E, González-Márquez H, Gómez-Olivares JL, Ortiz-Muñiz R (2008). Effects of moderate and severe malnutrition in rats on splenic T lymphocyte subsets and activation assessed by flow cytometry. Clin Exp Immunol.

[34] Testa U, Pelosi E, Peschle C (1993). The transferrin receptor. Crit Rev Oncog.

[35] Goral S (2011). The three-signal hypothesis of lymphocyte activation/targets for immunosuppression. Dial Transplant.

[36] Reicher B, Barda-Saad M (2010). Multiple pathways leading from the T-cell antigen receptor to the actin cytoskeleton network. FEBS Lett.

[37] Chávez-Galán L, Arenas-Del Angel MC, Zenteno E, Chávez R, Lascurain R (2009). Cell death mechanisms induced by cytotoxic lymphocytes. Cell Mol Immunol.

[38] Starling GC, Bajorath J, Emswiler J, Ledbetter JA, Aruffo A, Kiener PA (1997). Identification of amino acid residues important for ligand binding to Fas. J Exp Med.

[39] Sun M, Ames KT, Suzuki I, Fink PJ (2006). The cytoplasmic domain of Fas ligand costimulates TCR signals. J Immunol.

[40] Dzialo-Hatton R, Milbrandt J, Hockett RD, Weaver CT (2001). Differential expression of Fas ligand in Th1 and Th2 cells is regulated by early growth response gene and NF-AT family members. J Immunol.

[41] Gourley TS, Chang CH (2001). Cutting edge: the class II transactivator prevents activation-induced cell death by inhibiting Fas ligand gene expression. J Immunol.

[42] Carrington PE, Sandu C, Wei Y, Hill JM, Morisawa G, Huang T, Gavathiotis E, Wei Y, Werner MH (2006). The structure of FADD and its mode of interaction with procaspase-8. Mol Cell.

[43] Luo X, Budihardjo I, Zou H, Slaughter C, Wang X (1998). Bid, a Bcl2 interacting protein, mediates cytochrome c release from mitochondria in response to activation of cell surface death receptors. Cell.

[44] Li H, Zhu H, Xu CJ, Yuan J (1998). Cleavage of BID by caspase 8 mediates the mitochondrial damage in the Fas pathway of apoptosis. Cell.

[45] de Vries JF, von dem Borne PA, van Luxemburg-Heijs SA, Heemskerk MH, Willemze R, Falkenburg JH, Barge RM (2007). Differential activation of the death receptor pathway in human target cells induced by cytotoxic T lymphocytes showing different kinetics of killing. Haematologica.

